# Resolving the True Ventricular Mural Architecture

**DOI:** 10.3390/jcdd5020034

**Published:** 2018-06-20

**Authors:** Robert S. Stephenson, Peter Agger, Camilla Omann, Damian Sanchez-Quintana, Jonathan C. Jarvis, Robert H. Anderson

**Affiliations:** 1 Comparative Medicine Lab, Department of Clinical Medicine, Aarhus University, DK-8200 Aarhus, Denmark; robert.stephenson@clin.au.dk (R.S.S.); peter.agger@ki.au.dk (P.A.); camillaomann@clin.au.dk (C.O.); 2 Department of Anatomy and Cell Biology, Universidad de Extremadura, 06071 Badajoz, Spain; sanchezquintana55@gmail.com; 3 Research Institute for Sport and Exercise Sciences, Liverpool John Moores University, Liverpool L3 3AF, UK; j.c.jarvis@ljmu.ac.uk; 4 Institute of Genetic Medicine, Newcastle University, Newcastle upon Tyne NE1 3BZ, UK

**Keywords:** cardiomyocytes, ventricular walls, fibrous matrix, cardiac antagonism

## Abstract

The precise nature of packing together of the cardiomyocytes within the ventricular walls has still to be determined. The spiraling nature of the chains of interconnected cardiomyocytes has long been recognized. As long ago as the end of the nineteenth century, Pettigrew had emphasized that the ventricular cone was not arranged on the basis of skeletal muscle. Despite this guidance, subsequent anatomists described entities such as “bulbo-spiral muscles”, with this notion of subunits culminating in the suggestion that the ventricular cone could be unwrapped so as to produce a “ventricular myocardial band”. Others, in contrast, had suggested that the ventricular walls were arranged on the basis of “sheets”, or more recently “sheetlets”, with investigators seeking to establishing the angulation of these entities using techniques such as magnetic resonance imaging. Our own investigations, in contrast, have shown that the cardiomyocytes are aggregated together within the supporting fibrous matrix so as to produce a three-dimensional myocardial mesh. In this review, we summarize the previous accounts, and provide the anatomical evidence we have thus far accumulated to support the model of the myocardial mesh. We show how these anatomic findings underscore the concept of the myocardial mesh functioning in antagonistic fashion. They lend evidence to support the notion that the ventricular myocardium works as a muscular hydrostat.

## 1. Introduction

Any review of the “functional morphology” of the heart must provide an accurate account of the way in which the individual cardiomyocytes are aggregated together within the ventricular walls. It is the cardiomyocytes that are the working units of the myocardium. By their contraction, they provide the force for the ejection of the blood. Their precise arrangement within the walls, however, remains remarkably contentious. Advances in imaging techniques, nonetheless, are now permitting visualization of the transmural architecture in three-dimensions at ever increasing spatial resolution. This new level of morphological information suggests the myocardium is best described on the basis of a model which can be considered a three-dimensional myocardial mesh [1,2,3]. In spite of this detailed anatomical information, support continues to be provided for the notion that the ventricles are formed on the basis of a “ventricular myocardial band” [4,5]. Those supporting the concept illustrate their approach with models showing the band as a long thin structure extending as a wrapped entity between the pulmonary and aortic roots [6]. Others, in contrast, argue that the arrangement is one of ‘sheets’ [7], describing laminar arrays made up of four to six myocytes. Their studies using confocal microscopy [8] are compatible with the studies supporting the model based on the three-dimensional mesh [1,2,3]. Thus, their confocal images reveal a complex arrangement of aggregated cardiomyocytes with heterogeneous morphology. The aggregates themselves are embedded in an equally heterogeneous connective tissue matrix [9]. Others, using diffusion tensor magnetic resonance imaging to demonstrate the mural architecture, have described the arrangement in terms of smaller “sheetlets” within the wall [10,11]. It is difficult to match this alleged architecture with the existing morphological data. If these diverging approaches are to be reconciled, it will be necessary to show the precise manner of the aggregation of the individual cardiomyocytes within the ventricular walls, and to appreciate the extent and dimensions of the aggregated units. In other words, it will be necessary to define their boundaries.

**Figure 1:**
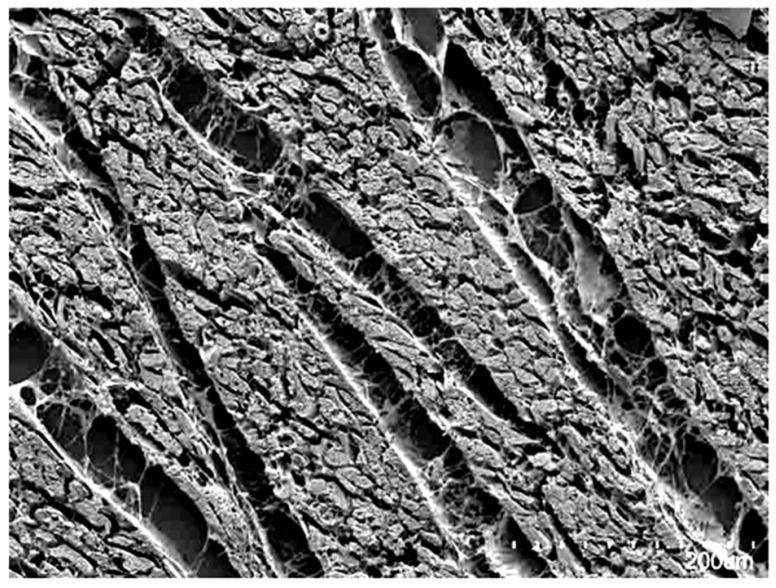
The scanning electron micrograph, taken from a sub-volume of the ventricular wall, shows how the individual cardiomyocytes are aggregated together by the endomysial component of the fibrous matrix into units that are separated by perimysial clefts containing loose connective tissue. There is, however, no uniformity in the thickness of the aggregated units, which can be seen to be heterogeneous and interconnected branching entities.

**Figure 2:**
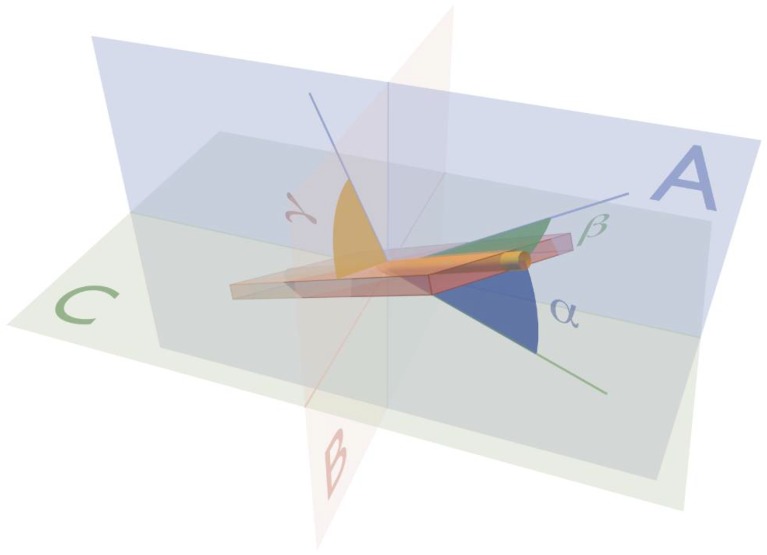
The drawing shows the angles used to define the three-dimensional orientation of the aggregated cardiomyocytes. Plane A is the circumferential-longitudinal plane parallel to the epicardium, while plane B is the radial-longitudinal plane. Plane B is parallel to the left ventricular long axis, and orthogonal to plane A. The third plane, C, is the circumferential-radial plane, which is orthogonal to planes A and B. The helical angle α is the angle between the chain of aggregated cardiomyocytes (yellow rod) and plane C. The transmural angle β is the angle between the chain and plane A. The E3 angle γ is measured as the angle between the aggregate and the circumferential-longitudinal plane. The unit of aggregated cardiomyocytes, depicted as a flat box, is a gross oversimplification of the true three-dimensional arrangement.

In this regard, it was established quite some time ago that each cardiomyocyte is supported by the endomysial weave of the mural fibrous matrix [12]. Perimysial clefts, containing loose fibrous tissue, were shown to interpose between the units of cardiomyocytes aggregated by the endomysial tissues (Figure 1). As already emphasized, these aggregated entities show remarkable structural heterogeneity within the different regions of the ventricular cone [13,14,15]. There can be no question concerning the presence of some degree of organization in the manner of their packing. This is because histological sections show obvious arrangements of the cardiomyocytes by virtue of local uniformities of their cross-sectional shape. The precise morphology, dimensions, and interconnections of the aggregated units, however, have yet to be described with certainty. Studies using microcomputed tomography and diffusion tensor magnetic resonance imaging [16,17,18], which support the model of the three-dimensional myocardial mesh, provide no evidence to support the concepts of aggregation on the scale that would be required to form a ventricular myocardial band. Instead, the three-dimensional orientation of these cardiomyocyte chains, and the aggregations they form, have been shown to be quantifiable at a much finer level, close to the size of individual cardiomyocytes. This permits the calculation of their helical and transmural angulations, and measurement of the so-called E2 and E3 angles (Figure 2, [11,19]). These findings, in turn, provide support for the notion that the complexity of the ventricular myocardial mass allows it to function in antagonistic fashion according to local demands [16]. The mural antagonism serves to preserve the ventricular shape, to store the elastic energy that drives the fast late systolic dilation, and to apportion mural motion so as to facilitate the spiraling nature of intracavitary flow [16,20].

In this review, we summarize again this anatomical evidence as collected by ourselves. More importantly, we present it in the context of historical evidence, some of which has been available for centuries. The pre-existing evidence itself has long served to challenge the notion that the ventricular myocardium can be analyzed in the fashion of skeletal musculature. These earlier findings negate the notion that the ventricular mass can be unwrapped as an anatomically traceable longitudinal band of muscle with both an origin and insertion [4,5]. The available evidence, along with new emerging findings, endorses the concept of a complex three-dimensional aggregation of the individual cardiomyocyte chains within a supporting fibrous matrix. The concept can be summarized in terms of “the myocardial mesh”. As yet, however, we do not have sufficient evidence to provide an accurate drawing illustrating the overall arrangement of the interconnections of the aggregated units across the thickness of the ventricular wall.

## 2. Previous Studies of Ventricular Mural Architecture

**Figure 3:**
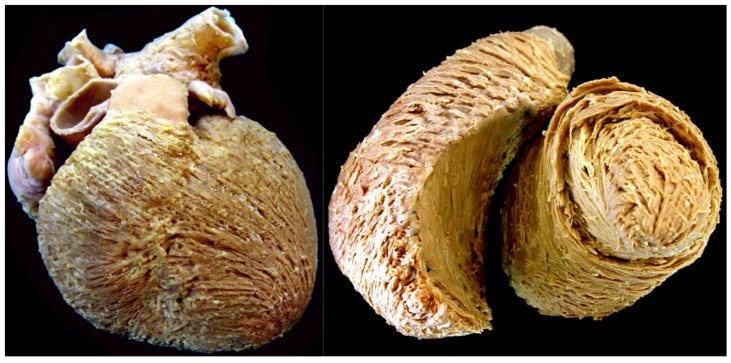
The dissection shown in the (**left hand**) panel was made by removing or ‘peeling’ the epicardial covering of the ventricular cone. It shows the obvious “grain” produced by the aggregation of the cardiomyocytes into chains. Note that the superficial cardiomyocytes are shared between the ventricles, with no traceable division. The left ventricular chains show a marked angle relative to the ventricular long axis. The image shown in the (**right hand**) panel demonstrates that the cardiomyocytes in the left ventricular mid-wall are aggregated in circumferential fashion.

The interest in myocardial architecture dates back hundreds of years. In Leonardo da Vinci’s drawings from the fifteenth century, we find notions on the varying alignment of the cardiomyocytes within the myocardium [21]. This was an idea that he allegedly adopted from Galen [22], having been proposed more than 2000 years ago [23]. The spiraling nature of the cardiomyocyte chains within the ventricular walls was also recognized and emphasized by Lower [24], and Sénac [25], in the seventeenth and eighteenth centuries respectively. They showed these details by gross dissection, a process which readily reveals the “grain” produced by the chains of individual cardiomyocytes (Figure 3).

It was such studies using gross dissection that formed the basis of the monumental studies of Pettigrew. Based on his dissections, Pettigrew had already determined that it was inappropriate to compare the arrangement of the cardiomyocytes within the heart with those of skeletal muscles [26,27]. As he stated, when summarizing his findings in his study published in three volumes “Unlike the generality of voluntary muscles, the fibers of the ventricles, as a rule, have neither origin nor insertion, that is, they are continuous alike at the apex of the ventricles and at the base” [28]. These salutary comments, however, were discounted by MacCallum [29], and by Mall [30]. It was they, who over the turn of the twentieth century, popularized the notion that the ventricular myocardial mass could be dissected so as to reveal sub-structures, which were described in terms of “bulbo-spiral muscles”, and similar entities. The validity of the dissections revealing such alleged independent components within the ventricular mass was then challenged in a careful study carried out by Lev and Simkins [31]. Grant reinforced their skepticism, stressing that entities could be sculpted from the ventricular myocardial cone at the whim of the dissector [32]. The dissections of Pettigrew, however, had shown that the “grain” produced by the aggregation of the individual cardiomyocytes changed with progression through the thickness of the ventricular wall. This spiraling nature of the aggregated cardiomyocytes was then quantitated by the studies of Streeter and his colleagues [33,34,35]. Two of these contributions were written in collaboration with Torrent-Guasp [34,35], albeit that one was published only in the form of an abstract [34]. In a remarkable summary of his studies, Streeter also provided an excellent review of the previous investigations [36], with his assessments being entirely in keeping with our own conclusions as described above.

It is perhaps surprising that, neither in his own detailed account [36], nor in the two publications produced in collaboration with Torrent-Guasp [34,35], no mention is made of the so-called “ventricular myocardial band”. It is, nonetheless, on the basis that the ventricular cone could be unwrapped to produce a band extending between the pulmonary and aortic roots that Torrent-Guasp himself, with his colleagues, described his findings in his final publication [5]. In contrast, Streeter [36] interpreted the dissections of Torrent Guasp as providing the validation of the concept advanced by Krehl at the end of the 19th century on the basis of the “triebwerk” [37]. To cite from this work [36], “In 1891, Krehl synthesized these constructs into a Triebwerk of the left ventricular wall minus the basal valve ring, apex, extreme epicardial fibers, and trabeculae. The Triebwerk was a nested set of fiber paths; each described figures of eight that decreased in amplitude on the inner sets. The innermost set was the ring of circumferential fibers.” In this regard, Streeter interpreted the “fiber” as representing the individual cardiomyocyte. As he commented [36], “From the work of Fox and Hutchins [38], it is evident that the cell is the proper anatomic unit of the myocardium. Any larger units of organization are separations of the interconnecting mass of cells by the vasculature and the connective-tissue stroma.” This statement is entirely in keeping with our own notion of the myocardial mesh. Streeter, in his monumental review [36], also discussed in detail the investigations of Pettigrew, to which we have already referred, but dismissing the notion that the cardiomyoctes were arranged in discrete layers. Instead, Streeter promoted the notion that, by tracing figure-of-eight “fiber paths”, the myocardial mass could be analyzed on the basis of nested toroidal, or geodesic, sets. He conceived the paths as being “characterized by a train of muscle cells on the epicardial side of the wall that spirals both down and into the wall, past mid-wall to the endocardial side, and then spirals up on the inside past the equator, where a similar passage past mid-wall returns the fiber train to the epicardial side of the wall.” In tracing such potential spiral paths, which were also described in both of the publications written in collaboration with Torrent-Guasp [34,35], he emphasized the necessity of the cardiomyocytes taking an “imbrication angle”. From his measurements, he concluded that “the transverse angle is small, negative and truly exists” [36]. This conclusion is important in itself, since conventional wisdom had been, and in some instances still is, based on the doctrine of Frank, namely that all cardiomyocytes were aggregated together in tangential, or surface parallel, fashion [39]. Indeed, in his chapter written together with Torrent-Guasp, he emphasized the point that the so-called “fiber paths” were not strictly tangential [35].

Streeter [36] also discussed at length the studies of Feneis [40], and Hort [41]. These investigators had prepared histological sections of the ventricular cone in short axis. They emphasized the “feathering” appearance created by the aggregation of the cardiomyocytes into interconnected units, findings again in keeping with our own observations [13]. It should be noted, however, that the ‘feathering’ observed in two-dimensional short axis sections can be somewhat misleading. Although consistent with the model of the myocardial mesh, this visual phenomenon tells us very little about the three-dimensional orientation of either the myocytes, or the units into which they are aggregated. The overall conclusion reached by Streeter [36], nonetheless, was that “The heart wall was shown to be a three dimensional continuum made up essentially of the one-dimensional rod element, the cardiac muscle cell.” It is impossible to reconcile these conclusions with the concept of the “ventricular myocardial band” as published in the final work of Torrent-Guasp and his colleagues [5]. This is of significance with regard to ventricular function, since those continuing to embrace the “band” concept view the different, and even the adjacent, components of the alleged band as having the capacity to function independently within the ventricular cone. It is also difficult to correlate the complex description of large scale “fiber pathways” as described by Streeter [36] with the current concept of small scale “sheetlets”, as put forward by some analyzing the ventricular cone using magnetic resonance imaging [10,11]. The detailed account provided by Streeter and his colleagues [33,34,35,36], furthermore, is exceedingly difficult to interpret with regard to the precise anatomical arrangement of his alleged “fiber paths”. Taken together, therefore, it seems that we still require an accurate, comprehensible, and appropriately illustrated account of the three-dimensional architecture of the ventricular myocardium. The three-dimensional imaging techniques now available to researchers will surely provide the necessary data to permit such an illustration to be provided in the very near future. At present, however, we lack the precise information required to illustrate the specific interconnections within the three-dimensional mesh, and the extent of the aggregated units of individual cardiomyocytes.

We can clarify the controversy as to whether all cardiomyocytes are aggregated together in tangential fashion. In this regard, Streeter had recognized the difficulty of measuring the true transmural orientation of the cardiomyocytes, which he named as the angle of imbrication, when using histological sections cut in the orthogonal planes of the ventricular wall [35]. He had sought to compensate for this problem by moving his block of tissue on a rotating chuck during the process of sectioning [36]. Other investigators similarly appreciated the difficulty of measuring the transmural angle. This angle is a non-projected version of the transverse angle. Another solution in providing this measurement is to cut full thickness blocks of the ventricular wall using circular knives [2,42]. The circular nature of the sections cut by the knives then compensates for the well-recognized changing helical angulation of the chains of cardiomyocytes, paralleling the changes made by Streeter using the rotating chuck. Measurements based on such sections obtained using circular knives, and stained histologically, however, have revealed far greater transmural angulation of the aggregated chains than described by Streeter. Only two-fifths of the measured chains of cardiomyocytes had angles within 7.5 degrees of the tangential plane. The remaining three-fifths of the chains deviated from the tangential plane by angles up to 45 degrees [2]. In a second study, also based on the sectioning of blocks from the walls using the circular knives, the sections obtained were images using diffusion tensor magnetic resonance imaging [42]. With this technique, the primary eigenvector of the diffusion tensor in each pixel was used to calculate the transmural angle of the long axis of the aggregated myocytes, relative to the epicardial surface. Of the approximately 2 million segments analyzed, 53% of the aggregated chains intruded or extruded transmurally by up to ±15°, 40% exhibited an angle of intrusion or extrusion of between ±15° and ±45°, while 7% exceeded an angle of ±45° [42]. These results are supported by another study conducted without the use of circular knives (see below).

These studies using circular knives have revealed the presence of chains of cardiomyocytes that deviate significantly from the tangential plane. The results parallel the conclusions of Streeter [34,35,36], albeit showing far greater degrees of intrusion or extrusion. The findings are in conflict with the doctrine of Frank, namely that all cardiomyocytes are aligned in tangential fashion. Measurements based on diffusion tensor magnetic resonance imaging, however, are based on the quantitation of spontaneous self-diffusion of water. They do not show the precise dimensions and structure of the chains and units into which the individual cardiomyocytes are aggregated. It is this deficiency that continues to underscore the ongoing controversies regarding the presence of “sheetlets”, and in determining their role in deformation of the ventricular walls during the cardiac cycle [11]. Thus far, quantification of the angulation of such entities has depended on the calculation of the secondary or tertiary eigenvectors [11,19]. The measurements made are unable in themselves to provide evidence regarding the dimensions and orientation of the aggregated units, since they cannot identify boundaries between one unit and another, should indeed such boundaries exist. Similar caveats apply to attempts to reveal the architecture of the aggregated units by anatomical dissection or histology. This is because, when the architecture of the ventricular myocardium is assessed using classic histologic techniques, the section chosen, be it longitudinal, horizontal, radial, or tangential, can reveal only two of the three orthogonal dimensions of the tissue studied. Attempts to circumvent this problem by reconstructing serial sections have thus far been non-productive, due to the inevitable distortions associated with the very process of histologic sectioning. It is possible that use of episcopic microscopy may circumvent this caveat. It is equally impossible to provide an accurate assessment of myocardial architecture using anatomical dissection, since some parts of the walls must be destroyed in order to display their deeper components. Even published accounts of the nature of the packing as revealed using synchrotron imaging have thus far proved disappointing, As stated by Teh and colleagues, “Resolution of sheetlet and sheetlet-normal orientations is complicated in SRI by the dense packing of cells, which can make it difficult to positively identify sheetlets” [43]. Whilst we recognize the obvious suitability of synchrotron imaging, our own preference thus far has been to use laboratory-based microcomputed tomographic imaging to reveal the anatomical arrangement of the packing of the cardiomyocytes, although as yet we have still been unable to distinguish the precise boundaries of the aggregated entities, should such boundaries exist.

## 3. Ventricular Mural Architecture as Revealed by 3-Dimensional Imaging

**Figure 4:**
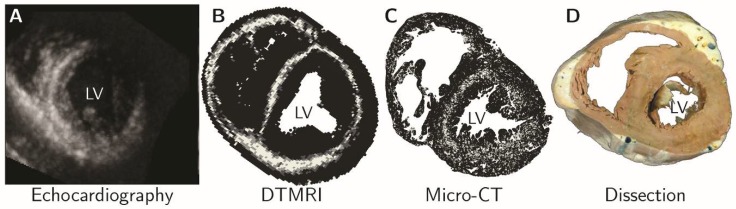
The morphological substrate for the enigmatic ‘echogenic bright line’ was resolved by multi-modality imaging. (**A**) The echogenic bright line viewed in the short axis derived from apical long axis images; Structure tensor analysis of diffusion tensor magnetic resonance imaging (**B**) and micro-computed tomography (**C**) data sets, white pixels indicate areas of circumferentially running myocytes; (**D**) This morphological arrangement it not easily appreciated in dissected specimens. Modified from Agger et al., 2016 [45].

Advances in the techniques now available for imaging autopsied specimens, such as micro-computed tomography and diffusion tensor magnetic resonance imaging, have changed the way we investigate cardiac architecture. We are no longer required to make assumptions based on quasi-three-dimensional techniques, such as dissection, peeling, or histology. Using so-called “eigen analysis” of the three-dimensional structural tensor [44], we can now extract information regarding the disposition of the cardiomyocytes in all three dimensions, quantifying their helical and transmural orientation, and measuring the angulation of the aggregated entities they form (see Figure 2). This high-resolution morphological information is now beginning to allow us to resolve the ongoing controversies regarding ventricular architecture. It permits us better to understand the intricacies of ventricular mural deformation in health and disease [17,18,19,45]. Such imaging of autopsied specimens has endorsed the characterization of the ventricular myocardium as a complex meshwork of heterogeneously arranged chains of cardiomyocyte. The chains are interconnected by side-branches, and supported within a network of connective tissue, thus producing a myocardial mesh [17,18]. None of this evidence supports the notion of the “ventricular myocardial band” [4,6]. Even those who initially supported the model now recognize its frailties [17,18,46]. Some phenomena explained on the basis of the band, furthermore, have been shown to have much better alternative explanations. Thus, Agger and his co-workers used multiple imaging modalities to clarify the substrate for the “echogenic bright line” [45]. This feature is observed in the apical long-axis view during echocardiographic examination (Figure 4).

**Figure 5:**
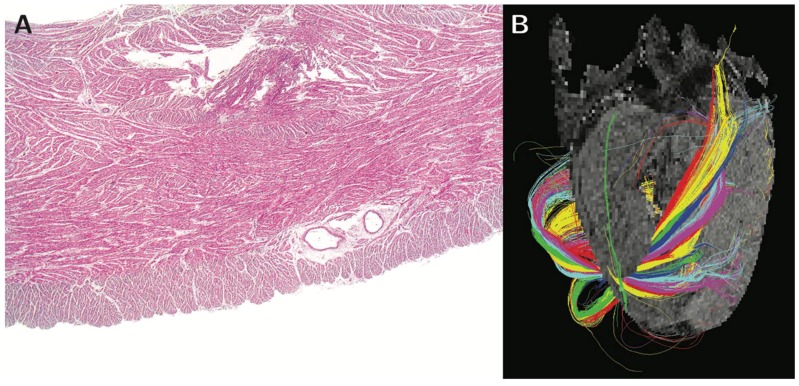
Panel (**A**) shows a histological section of the right ventricular wall of the sheep heart stained with hemotoxylin and eosin, revealing the heterogeneous mural architecture. The helical angle of the chains of cardiomyocytes aggregated together within the right ventricular walls is shown in panel (**B**), which is a tractograph generated from different zones of the walls. The color coding of the tractography does not represent any anatomical or physiological property. It serves only as a visual aid enabling the reader to distinguish between the tracks, and to visualize the mean orientation of cardiomyocytes within them. Modified from Agger et al., 2015 [48].

Those who continue to support the notion of the “myocardial band” had postulated that the ‘bright line’ represented a mid-septal space, allegedly interposed between the ‘limbs’ of the alleged band [47]. Analysis of high-resolution image data, in contrast, showed that the phenomenon reflects the helical orientation of the cardiomyocytes, specifically the interaction of the echo beam with the circumferentially arranged chains of cardiomyocytes aggregated together in the mid-wall of the septum (Figure 4). It is remarkable, therefore, that despite the evidence now provided by high-resolution interrogation of autopsied specimens (Figure 5, [48,49]), the very existence of such circumferential cardiomyocytes in the septum, along with the changing helical angulation of the chains of cardiomyocytes in the wall of the right ventricle, can continue to be questioned by supporters of the “band concept” [50].

**Figure 6:**
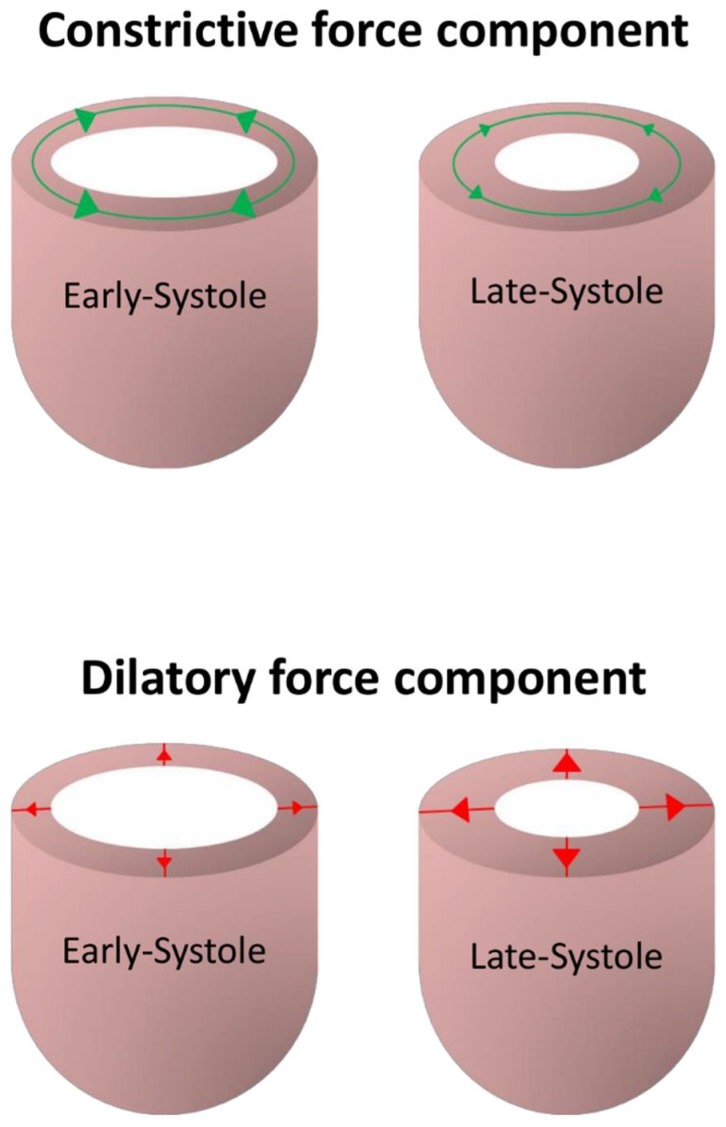
The drawings show the essence of mural antagonism. During contraction, tangentially arranged chains of cardiomyocytes are subjected to a progressive decrease in hemodynamic afterload. Because of this, their force magnitude decreases over the cardiac cycle, thus producing a constrictive force component which is unloading in nature. In contrast, during cardiac contraction, those chains of cardiomyocytes aligned in transmural fashion are subjected to a progressive increase in intrinsic afterload. Their force magnitude, therefore, increases over the cardiac cycle, thus producing an antagonistic and dilatory force component, which is auxotonic in nature. Modified from Lunkenheimer et al., 2018 [16].

Controversy has also continued regarding the presence of chains of cardiomyocytes with an orientation that deviates from the epicardial tangential plane, in other words those with a transmural orientation. Those denying the existence of such transmural aggregations seemingly ignore the evidence now provided by detailed three-dimensional imaging of autopsied hearts [51], which confirmed the findings obtained using circular knives [42]. They also fail to take note of the discussions provided by Streeter and his colleagues [34,35,36]. This again is of significance in understanding ventricular function. Thus, it was the recognition of the existence of cardiomyocytes aggregated in transmural fashion that underscored the re-emergence of a concept first described by Brachet [52], namely the notion of mural antagonism ([2,16,20], Figure 6).

Brachet’s concept [52], however, was shown to have no foundation in anatomic fact, since he had proposed separate existence of radial and tangential aggregated cardiomyocytes. When considered on the basis of the myocardial mesh, in contrast, it can be posited that those cardiomyocytes that are aligned in tangential fashion undergo a progressive decrease in hemodynamic afterload throughout cardiac contraction. Because of this, they exhibit a decrease in force magnitude over the cardiac cycle. This constrictive force, therefore, can be characterized as being ‘unloading’ in nature. The force vector created by the cardiomyocytes aligned in transmural fashion, in contrast, is dilative and auxotonic, in other words it increases during systole. Hence, it is predicted to act antagonistically with regard to ventricular constriction. This is because the aggregated cardiomyocytes aligned in transmural fashion undergo a progressive increase in intrinsic afterload as the wall thickens. Their force magnitude, therefore, increases across the cardiac cycle. As we learn more about how the components of the cardiac mesh are remodeled in disease, the concept of ventricular mural antagonism achieves increased clinical importance. From a diagnostic prospective, imaging studies suggest the transmural angle is remodeled in different disease states [19,48]. It is predicted that mural antagonism is increased in hypertrophy, and decreased in the setting of dilation. From a therapeutic point of view, furthermore, evidence already exists to show that the phenomenon can be selectively altered by administration of chronotropic drugs in a dose-dependent manner [53].

**Figure 7:**
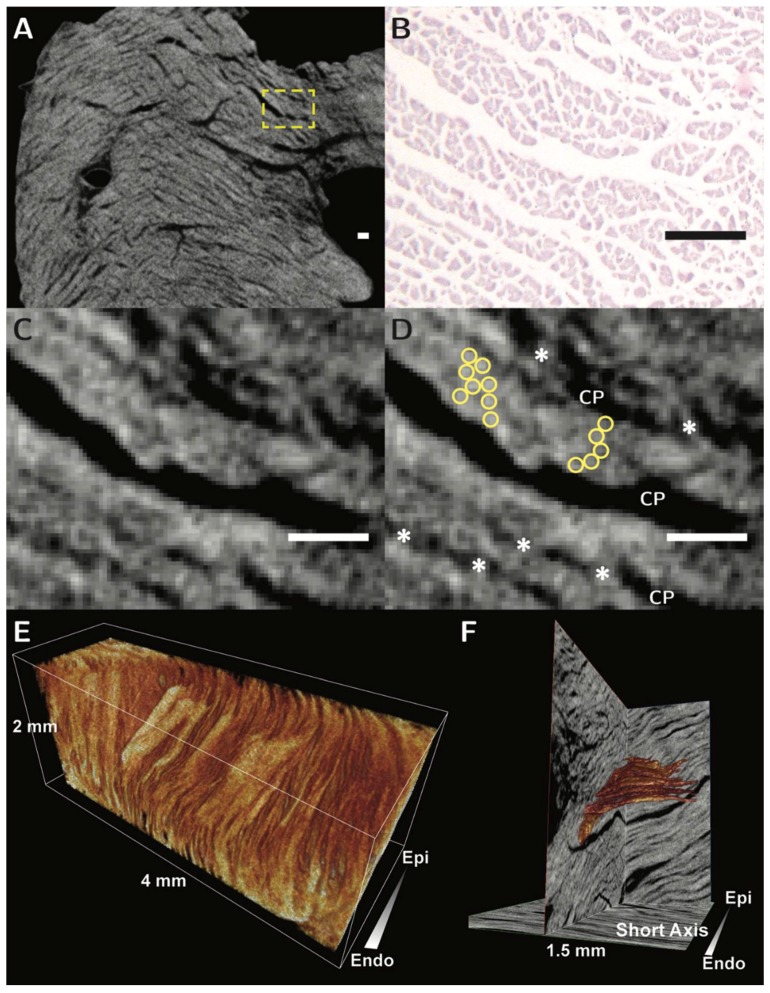
The figure shows the virtual histology of the aggregated units of cardiomyocytes as revealed using micro-computed tomography. Panel (**A**) shows a long axis transmural image obtained from the basal region of the posterior wall of the left ventricle. The arrangement of the cardiomyocytes in the yellow dashed box is then shown in panel (**B**), which is an histological section stained with hematoxylin and eosin, and panels (**C**,**D**), which are micro-CT images. It is possible, in panel (**D**), to recognize the individual cardiomyocytes, shown in the yellow circles, the branches between adjacent aggregated units, shown by the asterisks, and the cleavage planes (CP) between the units. The scale bars in panels B, C and D represent 100 µm. Panel (**E**) shows volume rendering of a subendocardial block of rabbit ventricular wall, showing the three-dimensional morphology of multiple aggregates. In Panel (**F**), a solitary aggregated unit of cardiomyocytes, shown in orange, has been reconstructed subsequent to resolution of the contained individual chains of cardiomyocytes. The illustrations are modified from Stephenson et al., 2016 [18]. Our work is now continuing so as to characterize the morphology of the cardiac mesh in different contractile states. Micro-computed tomography, therefore, now offers the means of providing three-dimensional validation of the exciting results already achieved in patients using diffusion tensor magnetic resonance imaging. When these studies are completed, we hope to be able to provide the much-needed accurate anatomical model of the three-dimensional mesh.

Recent studies using diffusion tensor magnetic resonance imaging have shown that re-orientation of the myocytes is not only crucial for normal contraction, but that the mobility of this process is altered in disease [11]. Thus, the orientation of the cells, and the units into which they are aggregated, is altered depending on the loading conditions to which they are subjected. In this regard, Nielles-Vallespin and associates, while assessing patients in the clinical situation using diffusion tensor imaging, had noted changes in the so-called “sheet angle” [11]. They characterized deformation of the aggregated units across the cardiac cycle in hearts of healthy patients, and those with hypertrophic and dilated hearts. They identified clear differences in the orientation and mobility of the aggregated units in the two disease sates. Such a technique has clear diagnostic potential, but requires validation in terms of the orientation and morphology of the alleged ‘sheets’. To date, studies of ventricular architecture in three or four dimensions have been validated only by using two-dimensional histology [15]. A method for three-dimensional validation is therefore desirable [43]. We have already shown the capability, using autopsied material, of contrast enhanced micro-computed tomography to resolve the alignment of these structures in three dimensions (Figure 7, [18]). We hope very soon to be able to provide the detailed account to show the three-dimensional architecture of the full thickness of the ventricular walls.

## 4. Functional Considerations

When assessing the ventricular mural architecture in the setting of ventricular function, it is pertinent to consider the concept of the muscular hydrostat. The basis of this notion is that distinct muscle bundles reduce one dimension of an enclosed fixed volume by their active contraction, thereby causing an increase in another dimension [54]. Such an arrangement is recognized within the skeletal muscles of the tongue, where it is the patterns of innervation that permit the longitudinal and transverse muscles to work independently. A comparable arrangement is found in the uvula, which is shortened by the force of its longitudinal muscles, but lengthened by the force of the circumferential muscles that wrap the structure. This functional arrangement is achieved thanks to the highly segmented innervation of its constituent muscles. The situation does not apply to the ventricular walls, which lack not only the dense compartmentation characteristic of skeletal muscular components, but also a lack of selective innervation of the myocardium. These features rule out the possibility that the heart can function in terms of a “ventricular myocardial band”, which looks to describe cardiac contraction based on innervation and deformation of distinct bands of muscle at specific points in the cardiac cycle. They do not, in contrast, mean that it is inappropriate to assess ventricular function on the basis of the muscular hydrostat. The function of the ventricular cone as a pump is based simply on the cyclical change in its endocardial surface, and the action of the valves. The volumes of muscles are almost constant during contraction and relaxation. The total volume of the ventricular cavities, therefore, changes as a function of the enclosed volume of blood [55]. It is on this basis that the mean mural thickening of around two-fifths achieved in systole is capable of generating a variation in ventricular luminal volume of almost two-thirds [56].

## 5. Conclusions

As we explore ventricular architecture with ever increasing elegance and resolution, it becomes increasingly apparent, at least in our opinion, that the most suitable description of ventricular architecture, and its correlation with ventricular function, is provided by the model of the myocardial mesh. The findings described in our review are not themselves new, although we are currently expanding them using micro-computed tomography. We consider it necessary to bring our findings to the forefront once more. This is because, despite the existence of much evidence already negating the concept, some continue to argue in favor of the ventricular cone being arranged on the basis of a “ventricular myocardial band” [50]. As emphasized above, the notion of the “myocardial band” is incompatible with the concept of either the muscular hydrostat or the three-dimensional mesh. The functional unit of the myocardium is the cardiomyocyte itself. It is then the complex three-dimensional orientation of the units into which they are aggregated, along with their specific loading conditions, which dictate the efficacy of mural thickening. High-resolution morphological studies are beginning to show how the components of the myocardial mesh deform in both healthy and diseased hearts. They will constitute the important next step in elucidating the mechanisms of ventricular function. This information will surely highlight potential therapeutic and surgical targets, and provide three-dimensional validation of ongoing clinical imaging of the ventricular mural architecture.
